# A review of the effects of dietary organic acids fed to swine

**DOI:** 10.1186/s40104-015-0042-z

**Published:** 2015-10-21

**Authors:** Mocherla V A N Suiryanrayna, J V Ramana

**Affiliations:** Livestock Research Station, Sri Venkateswara Veterinary University, Garividi- 535 101, Vizianagaram District, Andhra Pradesh India

**Keywords:** Chelation, Improved productivity, Organic acids, Pathogen inhibition, Protein utility, Swine

## Abstract

Animal production depends on nutrient utilization and if done there is an accelerated momentum towards growth with a low cost to feed ratio Public concern over the consumption of pork with antibiotic residues of the animals fed with antibiotic growth promoters (AGP) has paved the way to use other additives like herbs and their products, probiotics, prebiotics etc. Numerous feed additives are in vogue for achieving this target and one such classical example is the usage of organic acids and their salts. Usage of organic acids was in progress for over four decades. Early weaned piglets are (3–4 weeks age) exposed to stress with a reduced feed intake, little or no weight gain. This post weaning lag period is due to a limited digestive and absorptive capacity due to insufficient production of hydrochloric acid, pancreatic enzymes and sudden changes in feed consistency and intake. Lowering dietary pH by weak organic acids was found to overcome these problems. The main activity of organic acids is associated with a reduction in gastric pH converting the inactive pepsinogen to active pepsin for effective protein hydrolysis. Organic acids are both bacteriostatic and bactericidal. Lactic acid has been reported to reduce gastric pH and delay the multiplication of an enterotoxigenic *E. coli.* These acids are the intermediary products in Kreb’s cycle and thus act as an energy source preventing the tissue breakdown resulting from gluconeogenesis and lipolysis. Excretion of supplemental minerals and nitrogen are minimized with organic acids as these form complexes with minerals and aids for their bio-availability. Short chain fatty cids like acetic, propionic and n-butyric acid produced by microbial fermentation of dietary fibre in the large intestines may increase the proliferation of epithelial cells and have stimulatory effects on both endocrine and exocrine pancreatic secretions in pigs. Organic acids also enhances apparent total tract digestibility and improves growth performance. It is concluded that organic acids and their salts increase the protein utilization especially in weaner pigs and improves production indices.

## Background

When the basal liquid milk diet is reduced and the stage where in the piglets turn into solid diet of creep ration, the digestive physiology changes where in the intervention of different feed additives is needed to have the maximum nutrient utilization. Gastro-intestinal disturbances during pre and post weaning conditions cause large economic losses in pig industry. The weaning transition is a complex period during which the piglets have to cope up with abrupt separation from their mother, mixing with other litters in a usually new environment and turning over to a less digestible solid creep feed to highly digestible liquid milk. The situation remains same when the new born piglets are offered with solid creep feed from the 10th day after birth. Weaning exacerbates the level of general stress in these immature animals [1].

It was reported that 50 % of weaned piglets consume their first meal within 24 h post weaning, 10 % have not eaten until more than 48 h [2]. and thus the energy requirements for maintenance are only met 3 days post weaning and it takes 8–14 days for piglets to recover their pre-weaning level of energy intake [3].

Intensification of swine rearing paved the way for better productive performance and this has led to the shortening of the suckling period of piglets from the usual 6 weeks to 3–4 weeks of age in order to maximize annual sow productivity. A complication of early weaning leads to post weaning diarrhoea, which causes retarded growth, increased mortality [4]. In order to check the diarrhoea and improve the performance, prophylactic doses of antimicrobial feed additives like antibiotics, and antibiotic like growth promoters are being added to weaner and grower diets. Antibiotics have been used in animal production for over 50 years. Feeding of antibiotics was very successfully adopted and has become an integral point of developing nutritional strategies for all farm animals [5]. Feeding swine with antibiotics has been documented to increase weight gain by 3.3–8 % and improves feed efficiency approximates by 3 % [6]. In recent years public concern over the use of antibiotics which led to the development of resistant pathogen strains and antibiotics residues in animal products and these consequences encouraged to search for alternative means of controlling scours with no hazard to humans.

There was an increased awareness on the ban of using antibiotics to avoid their residues in meat and about the risk developing cross-resistance of pathogens to antibiotics [7, 8].

In order to prevent bacterial resistance against antibiotics, like other feed additives, fermented liquid feed (FLF) has been suggested as alternatives [9]. FLF not only reduces the gastric pH but gives simultaneous provision of feed and water which may result in easier transition from sow’s milk to solid feed for the piglets [10, 11].

FLF is produced by incubating the feed together with either the water or by product from the food or ethanol production. During the period of incubation fermentative micro organisms produce different organic acids, mainly lactic acid and acetic acid which reduce the gut pH [12]. Because of it’s probiotic properties, FLF is proved better than organic acids. A well fermented feed with lactic acid bacteria is a very cost-effective method to produce organic acids.

Organic acids can be both bacteriostatic and bacteriocidal and these actions depend on the levels of their inclusion. These acids can be effectively used along with other feed additives [13].

Various other feed additives such as organic acids, copper sulphate, zinc oxide, probiotics, prebiotics and herbs have been studied in newly weaned piglets [14–16]. Partanen and Morz Z [17] reported that the inclusion of organic acids in the diet can enhance growth performance and modulate intestinal microbiota in pigs. Lactic acid has been reported to reduce gastric pH and delay the multiplication of an enterotoxigenic *E. coli* [18] and to be more effective than other organic acids in improving pig growth performance [16].

When the piglets are weaned earlier at 3–4 weeks of age, they are exposed to both nutritional and environmental stress which often results in reduced feed intake, little or no weight gain and in some instances diarrhoea, morbidity and death. This postweaning lag period is a result of a limited digestive and absorptive capacity due to insufficient production of hydrochloric acid, pancreatic enzymes and sudden changes in feed consistency and intake [4, 19]. At this age the immunological status of a piglet is also low as passive immunity acquired through maternal colostrum is dramatically decreased, and active immunity is only beginning to develop [20]. Lowering dietary pH by weak organic acids, such as citric, formic, fumaric, lactic or propionic acids has been reported to be helpful in overcoming problems of the post weaning lag period [21, 22].

Acidifying the feed or water has started way back in 1968 as [23] added 0.8 % lactic acid to drinking water and reported that the growth response and feed efficiency in weaning piglets were significantly improved with a reduction in the haemolytic *E. coli* counts both in the duodenum and jejunum. Earlier reports [24–26] documented beneficial effects on performance of weaning pigs by adding organic acids or acidifiers. In the present context, the word organic acid is a pure acid and acidifier includes organic acid salts also.

A low pH in the gut is beneficial in several ways. It will increase the activity of the enzyme pepsin which enhances the utilization of the protein which is good both for the environment and economy of production. Low pH also increases the digestibility of nutrients through the changes in the villus height and depth in the small intestines in young piglets. This phenomenon can be explained as the protein from milk (casein) needs the pig’s stomach to have a pH of 4 in order to coagulate, precipitate and reach a maximum digestibility of about 98 %. But the case of vegetable and fish proteins is different which needs pepsin for maximum efficiency at a desired pH of 2–3.5 which is only achieved by organic acids.

Although the organic acid supplementation was initially targeted for weaned piglets, there is growing evidence that dietary acidification may also be beneficial for fattening pigs. The apparent ileal digestibility of protein and amino acids [27–29] and absorption of minerals [30] were improved in fattening pigs by the addition of organic acids. This may contribute not only to improved performance but also to reduced Nitrogen and Phosphorus excretion with decreased environmental pollution. Organic acids are also known as effective preservatives which protect stored feeds against undesirable bacterial or fungal growth [31], and improved quality of feeds over time may further contribute to improved performance. The main action by which acidifiers store feed ingredients is by the way of reducing the pH of the feeds [17].

The aim of this review is to evaluate the response of weaned piglets, growing pigs and reproductive m sows to dietary organic acids as illustrated in terms of performance, i.e., growth rate, feed intake and feed utilization. In addition, reasons for varying responses to and possible modes of action of organic acids will be discussed.

## Methodological review

### Mode of action of organic acids

Like antibiotics, organic acids have an antimicrobial activity. The acids can penetrate the bacterial cell wall and disrupt the normal actions of certain types of bacteria including *Salmonella* spp, *E. coli*, *Clostridia* spp, *Listeria* spp. and some coliforms. Therefore, reduction in numbers of some species of the normal intestinal bacteria as well as pathogenic bacteria can occur in animals fed organic acids. Organic acids are believed to improve overall performance by reducing microbial competition with the pig for nutrients, by lowering the risk of subclinical infections, reducing the intestinal immune response and by reducing the production of harmful bacterial compounds. In a nut shell organic acids lowers gastric pH [26, 32], converts inactive pepsinogen to active pepsin, inhibits pathogenic bacteria proliferation, acts as an energy source in GI-tract, aids in gastric emptying rate enhances endogenous enzyme secretion and chelates minerals [33] which are discussed here under.

#### Lowering of stomach pH

The main action of organic acids in poultry is mainly antimicrobial, whereas in pigs, a key activity is reduction of stomach pH [34]. In the pig, protein digestion begins in the stomach with the action of pepsin, secreted as the enzyme precursor, pepsinogen by stomach mucosa. Conversion of pepsinogen to pepsin occurs rapidly at pH 2.0 but only slowly at pH 5.0 to 6.0. In turn, pepsin works best in an acidic environment, pH 2.0 to 3.5, and activity declines rapidly above this pH. Carbohydrate hydrolysis in the stomach occurs by the action of salivary amylase, which, in contrast to pepsin, is inactivated once pH falls to 3.5.

In the suckling pig, acid secretion is low and the principal source of acidity is bacterial fermentation of lactose from sow’s milk to lactic acid. A high level of lactate in the stomach tends to inhibit HCl secretion. Ingestion of solid feed reduces the level of lactic acid in the stomach and stimulates HCl production but, in practice, creep feed consumption is low and variable at least up to 4 weeks of age [35]. At weaning, a combination of low acid secretion, lack of lactose substrate, and consumption of large meals at infrequent intervals can result in elevated pH, often to over 5.0 and it may remain high for several days. The high acid-binding/buffering capacity of the feed helps to further raise the stomach pH. Inclusion of whey or lactose in the starter diet ensures continuation of bacterial fermentation and lactic acid production. Development of HCl secretory capacity occurs more rapidly in the weaned pig than in the suckling pig. [36] reported a reduction in stomach pH from 4.6 to 3.5 by the addition of 1 % citric acid and from 4.6 to 4.2 by 0.7 % fumaric acid in the diet. On the other hand inorganic acids, such as hydrochloric or phosphoric acid (both of which reduce stomach pH), do not improve growth rate or feed conversion of pigs *in vivo* [37].

Lowering the acid-binding capacity of diets for newly-weaned pigs can help ease the transition from milk to solid food at weaning. Raised stomach pH after weaning results in reduced digestion of feed which will then be fermented in the hind gut and may provoke diarrhoea. A high gastric pH will also allow pathogens to survive and allow them greater opportunity to colonise the digestive tract [38].

#### Inhibition of pathogenic bacteria

Lactic acid has been reported to reduce gastric pH and delay the multiplication of an enterotoxigenic *E. coli* [18] and to be more effective than other organic acids in improving pig performance [16].

Shift from milk diet to solid creep diet in weanling piglets is known to disturb the intestinal microflora balance and may adversely affect the gastro-intestinal functions [39]. It is well known that low pH in association with rapid flow of digesta can reduce the colonization of microbes in the gastro –intestinal tract [40].

As a matter of fact, animals and plants live in symbiosis with different bacteria, which can protect the host from the colonisation of pathogenic bacteria, regulate the development of the gut or produce vitamins and hormones for the host, while some bacteria are also known to cause diseases. However, the presence of bacteria within the gastro-intestinal tract in general also leads to the competition of the host animal and the bacterial population for nutrients. Bacteria furthermore secrete toxic compounds i.e., toxic amino acid catabolites, decrease fat digestibility, stimulate rapid turnover of absorptive epithelial cells, require an increased rate of mucus secretion by intestinal goblet cells and stimulate immune system development and inflammatory responses. All of these effects lead to impaired growth performance and research has demonstrated that as much as 6 % of the net energy in pig diets is lost to the microflora.

Therefore, it is not only highly important to control possibly harmful bacteria, but also to keep the bacterial population within the gut well balanced. Already a long time ago organic acids were identified to be able to alter the gastro-intestinal microflora by reducing in particular acid-intolerant bacterial species such as *E. coli, Salmonella* and *Campylobacter* resulting in increased growth performance. However, it was also shown, that organic acids have stronger effects in the inhibition of gram-positive bacteria. This is due to the structural differences of gram-positive and gram-negative bacteria. In general, the cytoplasm of the cell is surrounded by the cytoplasmic membrane. The cytoplasmic membrane is covered by a thick cell wall layer mainly consisting of peptidoglycan and adjoined by extracellular polysaccharides, teichoic acids and teichuronic acids. The peptidoglycan layer is significantly thinner in gram-negative bacteria compared to gram-positive bacteria. However, gram-negative bacteria are surrounded by an additional outer membrane which provides the bacteria with an inherent resistance to hydrophobic antibiotics and detergents due to the presence and features of lipopolysaccharides in the outer membrane.

Often organic acids were combined with other naturally derived products such as essential oils in an attempt to use possible synergism to combat pathogenic bacteria. Essential oils in general serve as antioxidants, stimulate the immune system, suppress harmful microorganisms on one side, but stimulate beneficial microbes on the other, by regulating the activity of enzymes especially lipase, which protects the gut villi and interferes with the DNA replication of bacterial cells and therefore have anti-bacterial effects.

**Fig 1:**
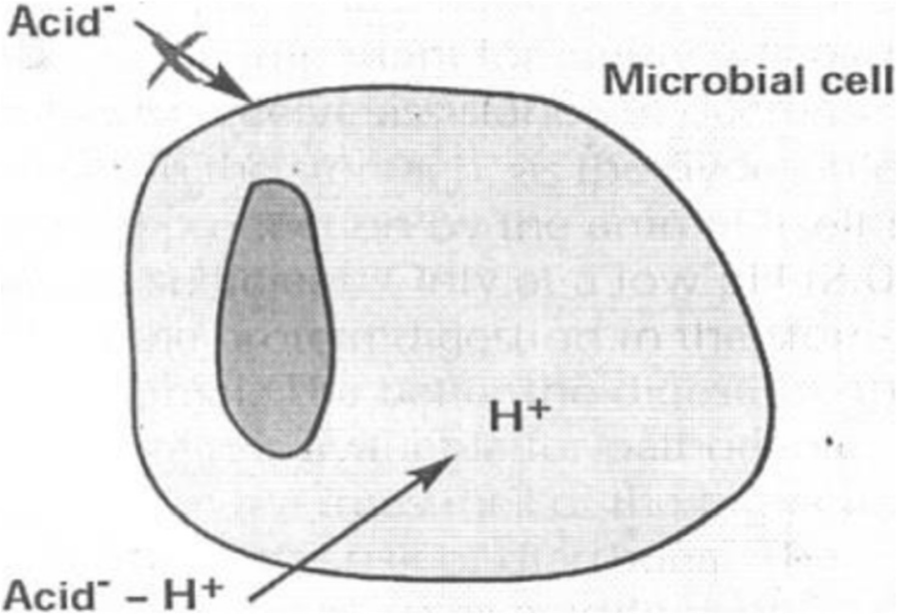
Only the not dissociated acid penetrates the microbial cell membrane

When the piglets face a stress on shifting of liquid to solid feed and during weaning, if the stomach pH is not lowered, coliforms dominate with a reduction in *Lactobacilli* [41]. It was reported by [42] that acidic conditions in the stomach favour the growth of *Lactobacilli* which inhibits the colonization and proliferation of *E. coli* by blocking the sites of adhesion or by producing lactic acid and it’s metabolites which lower gastric pH and thus checks the pathogens. More over organic acids have strong bactericidal properties. Non-ionized organic acids can penetrate the bacterial cells and disrupts the normal physiology of the bacteria [43]. When the undissociated organic acids (Fig. 1) penetrate the bacteria, they get dissociated to H^+^ and anions (A^−^). This action further reduces the internal pH of the bacteria checking the growth of pH sensitive Coliforms, *Clostridia*, *Listeria* because these bacteria cannot tolerate the broad range of internal pH in the bacteria and external stomach pH. On the other hand, the non-pH sensitive bacteria like *Lactobacilli* and *Bifidibacterium spp.* can tolerate [44] these variations in the internal and external pH. Non-dissociated organic acids are not absorbed by the intestinal epithelium.

Organic acids are both bacteriostatic and bactericidal. As undissociated organic acids are lipophilic, they can cross the cell membrane of gram negative bacteria, such as *Salmonella*. Once inside the cell, the higher cytosolic pH causes the acid to dissociate, releasing hydrogen ions, which consequently reduces the intracellular pH. Microbial metabolism is dependent on enzyme activity, which is depressed at lower pH. To redress the balance, the cell is forced to use energy to expel protons out across the membrane via the H^+^ - ATPase pump to restore the cytoplasmic pH to normal. Over a period of exposure to an organic acid, this can be sufficient to kill the cell [13].

It was reported that acidifiers improve gut health by promoting the beneficial bacterial growth while inhibiting the growth of pathogens through the reduction of pH and buffering capacity of the diets. The reduced buffering capacity of the diets containing organic acids reduces the colonization of undesirable microbes [45–48].

*Salmonella enteritica* Typhimurium is the predominant serotype found in pig carcasses in Europe, accounting for about 71 % of cases. Several serotypes are resistant to antibiotics, which has put pressure on producers to prevent contamination. While *Salmonella* cannot be wholly eradicated in pig units, it can be controlled to minimise the risk to consumers. Although heat treatment is effective in reducing contamination of feed leaving the feed mill, this effect does not persist during transport, storage and subsequent out feeding. When conditions within the feed are less conducive to bacterial infection, *Salmonella* contamination can be reduced. The next critical control point is within the pig’s gut, where conditions for bacterial growth may again be optimal. *Salmonella* growth is favoured at moisture content greater than 12 % and a pH between 4.5 and 9.0. It is no coincidence that the pig gut can provide *Salmonella* everything they need to thrive [49].

#### Energy source

Organic acids act as an energy source in the gut of pigs as these are the intermediary products of tricarboxylic acid and thus helps in preventing the tissue breakdown resulting from gluconeogenesis and lipolysis [50]. It was reported by [32] that the growth promoting effects of the organic acids were due to their energy values. Kirchegessner and Roth [51] suggested that pigs can utilise fumaric acid as energy source as efficient as glucose. Blank et al. [52] reported that there is a possibility that fumaric acid as a readily available energy source may have a local trophic effect on the mucosa in the small intestines and lead to an increase in the absorptive surface and capacity in the small intestines due to faster recovery of the gastro-intestinal epithelial cells after weaning.

#### Mineral utilization

Organic acid anions can complex with calcium, phosphorus, magnesium and zinc, improving the digestion of these minerals and reducing the excretion of supplemental minerals and nitrogen. The effects of organic acids on phytate P utilization might result from a change to the pH of the gastrointestinal tract to a pH more favorable for phytase to hydrolyze phytate [53]. Kirchegessner and Roth [51] reported that the apparent absorption and retention of Calcium, Phosphorus and Zinc were improved by the addition of fumaric acid. A decrease in intestinal pH is favourable for the P solubility [54, 55] and it was reported that the microbial phytase is more active at lower pH and thus addition of organic acids indirectly helps in P absorption. Boling et al. [56] suggested that citric acid improved phytate P utilization by competitively chelating Calcium, reducing the formation of insoluble Ca-phytate complexes. The intensity with which the organic acids work depends upon the type of diet and the dietary mineral content. In pigs fed with suboptimum levels of Zinc, addition of 15 g of citric acid per kg feed did not show the symptoms of parakeratosis [57, 58] but no significant effects on the apparent absorption and retention of zinc or other minerals (Ca, P, Mg, Fe, Cu and Mn) were found.

#### Endogenous enzyme secretion and gut morphology

It was reported that short chain fatty acids have stimulatory effects on both endocrine and exocrine pancreatic secretions in pigs. The natural acids like HCl in the stomach can get a pH of 1.3 where as the lactic acid produced from lactose in sow’s milk is able to produce a pH of 3.8 [59]. Above this pH, serum secretin levels decrease. Intestinal acidification either with Hcl or monocarboxylic acids or organic acids elevates serum secretin content. Both pancreatic exocrine secretion and biliary excretion are stimulated by the release of secretin [59]. Pancreatic exocrine response by monocarboxylic acids was highest for formic acid followed by lactic acid, pyruvic acid, acetic acid, butyric acid and propionic acid. As shown by [60, 61], short chain fatty acids such as acetic, propionic and n-butyric acid produced by microbial fermentation of dietary fibre in the large intestines may increase the proliferation of epithelial cells. Overland et al. [62] demonstrated an increase in the length of the microvilli in the ileum and the depths of the crypts in the caecum in growing pigs when fed with 0.17 % of Sodium butyrate and thus the gut morphology is changed. Increased epithelial cell proliferation has also been observed when short chain fatty acids are given orally or by intravenous injections or gastro-intestinal infusion [61] as dietary organic acidifiers can influence fermentation patterns in the small intestines. Other studies have demonstrated that addition of dietary organic acids in pigs stimulates secretion via metabolic enzyme activity. Butyric acid for instance, is the main energy source for the epithelial cells of the large intestine and is considered to be effective for promoting epithelial growth [62].

#### Performance and nutrient utilization

Effective dietary doses of organic acids have been established [63] that can improve productivity of pigs to levels comparable with antibiotic growth promoters. Overland et al. [64] added 0.8 or 1.2 % potassium diformate to diets for primiparous and multiparous sows from one mating lactation. The performance of the piglets of these sows was also recorded and compared. The authors found that sows fed potassium diformate had increased back fat thickness during gestation, although daily feed intake and body weight gain did not change. Feeding potassium diformate also tended to be associated with a heavier birth weight of piglets, irrespective of dose. It also improved average daily gain, resulting in a greater weaning weight. Sows fed the diets containing potassium diformate tended to have increased milk fat content on day 12 post-farrowing. On the other hand, sows fed potassium diformate at a dosage of 0.8 % under tropical conditions [65] tended (*P* < 0.1) to have a higher feed intake from 3 days after farrowing. Furthermore, reduced weight loss (*P* = 0.06) during the weaning period and lower back fat loss (*P* = 0.05) was observed.

**Fig 2:**
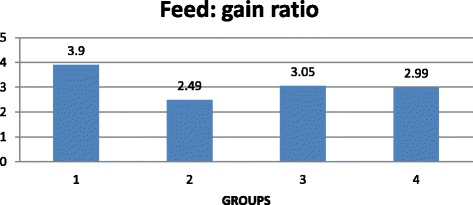
Effect of citric acid on feed: gain ratio of pre-weaned cross-bred piglets [Source: [66]; Suryanarayana et al.]

**Fig 3:**
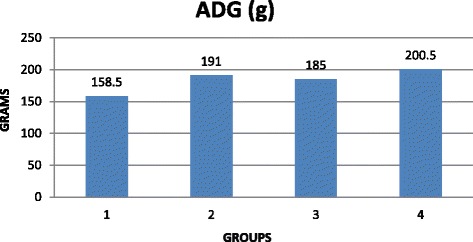
Effect of citric acid on ADG (g) in pre-weaned cross-bred piglets [Source: [66]; Suryanarayana et al.]

Addition of sodium format to grower diets at 0.9 % improved [67] the ADG (g) and feed: gain ratio (*P* < 0.05) but not in finisher pigs. They reported that CP and DM digestibility were higher (*P* < 0.05) for grower cross-bred pigs supplemented with 0.9 % sodium format. Falkowski and Aherne [21] demonstrated that ADG (g) was 4 to 7 % greater and feed conversion ratio was also improved 5 to 10 % when fumaric or citric acid was provided to pigs weaned at 4 weeks age. Giesting and Easter [50] reported that addition of graded levels of fumaric acid at 0, 1, 2 3 and 4 % resulted in a linear increase in gain:feed, daily gain regardless of dietary protein level. It was reported by Blank et al. [52] that dietary inclusion of fumaric acid improved the ileal digestibility of CP, gross energy and some amino acids. On the contrary, [68] reported a negative effect on ileal digestibility of crude protein and amino acid with increasing levels of fumaric acid supplementation in wheat-soybean meal based rations in pigs. It was demonstrated [66] that dietary supplementation (Figs. 2 and 3) of 0.15 % of citric acid to corn-soybean meal based rations improved (*P* < 0.05) ADG (g) and weight gain (Table. 1) with a non-significant feed: gain ratio in pre-weaned piglets. Metzler and Mosenthin [37] reported different apparent total tract digestibility of crude protein and energy (Table 2) and on nitrogen (N) retention in pigs fed with various organic acids. Increased proportion of organic acids disturbs acid base balance, metabolic acidosis and decreased feed intake [29] with a reduced performance.

**Table 1:** Effect of citric acid on growth performance of pre-weaned cross-bred piglets (Source: [66])

Parameter	G_1_	G_2_	G_3_	G_4_
Initial weight, kg	4.20 ± 0.05	4.87 ± 0.59	4.78 ± 0.29	3.57 ± 0.09
Final weight, kg	13.40 ± 0.88	13.55 ± 0.81	13.74 ± 0.88	13.92 ± 1.39
Total weight gain, kg	9.04 ± 0.11^b^	12.42 ± 0.07^a^	11.52 ± 0.56^a^	11.99 ± 0.32^a^
ADFI, g	652 ± 2.83^a^	628.5 ± 4.59^a^	595.5 ± 4.59^b^	555 ± 8.49^c^
ADG, g	158.50 ± 1.77^b^	191 ± 8.48^b^	185 ± 2.83^b^	200.5 ± 3.18^a^
Feed: gain ratio	3.90 ± 0.65	2.49 ± 0.1	3.05 ± 0.83	2.99 ± 0.71

G_1_- Control; G_2_- Citic acid (0.15 %); G_3_- Probiotic (0.1 %); G_4_- Citic acid (0.15 %) & Probiotic (0.1 %)

**Table 2:** Effects of organic acids on the apparent total tract digestibility of crude protein and energy and on nitrogen (N) retention in pigs (Source: [37])

	Level	Crude protein		Gross energy		N retention	
Organic acid	(%)	D (%)	ΔD	D (%)	ΔD	R (%)	ΔR
Formic acid	1.4	80.6	+1.4	82.2	+0.7	48.3	+4.9*
Butyric acid	2.7	80.6	+1.4	82.2	+1.6*	48.3	+4.0*
Formic acid	1.8	80.6	−1.0	82.2	+0.7	48.3	+2.9*
Propionic acid	2.0	80.2	+2.3	77.9	+1.4	-	-

*D* digestibility of non-acidified control diet, *ΔD* percentage unit change in the digestibility relative to the non-acidified control diet, *R* N-retention of non-acidified control diet as a percentage of intake, *ΔR* percentage unit change in N-retention relative to the non-acidified control diet

*significantly different from the control diet (*P* < 0.05)

### Beneficial effects of organic acids on swine

The anti microbial activities of organic acids differ from acid to acid depending upon concentration and pH. As examples, Lactic acid is more effective in reducing gastric pH and Coliform [16, 69, 70, 71] whereas other acids like formic and propionic acids have broader range of activity on *Salmonella*, Coliforms and *Clostridia* along with fungi and yeast [17, 32, 71, 72] and are not specific. Several reports have indicated that organic acids reduce the coliform population in the gut and reduce scouring in piglets and also control post-weaning diarrhoea [47, 73].

### Microbial adaptation to acids

Tolerance to acidic environment is recognised as an important survival strategy for many microorganisms. Recent developments in understanding this phenomenon include the identification of regulatory, as well as structural genes, involved in specific tolerance mechanisms. The unifying concept is that the microorganism under siege will sense a deteriorating environment and undergo a programmed molecular response by which specific, stress-inducible proteins are synthesised. These proteins presumably act to prevent or repair macromolecular damage caused by the stress. Some stress proteins are induced by a range of stress conditions, whereas others are induced in response to a specific stress [74]. According to [74], correlation exists between the response of enterobacteria to acid stress and pathogenecity. Kwon and Ricke [75] suggested that SCFA in the gastrointestinal tract of a host animal or in food materials might contribute to the enhancement of the virulence of *S. typhimurium* by increasing acid tolerance. Studies on acid adaptation mechanism of *Streptococcus mutants* [76] showed that growth at pH 5 resulted in significant changes in membrane fatty acid composition compared with cells grown at pH 7. According to these authors, the shift in the unsaturated/saturated ratio with growth at lower pH suggests that changes in membrane fatty acid composition are directly related to the acid adaptive response.

## Individual organic acids in animal nutrition

### Citric acid

Citric acid is colourless and crystalline with a pleasant sour taste. This is less anti-bacterial as compared to other acids. Adding 1.5 % citric acid to control diets did not significantly affect pH, the concentration of volatile (VFA) or non-volatile fatty acids (NVFA), or microflora (total anaerobes, *Lactobacilli, Clostridia*, *E. coli*) in the contents from the stomach, jejunum, caecum or lower colon of weanling pigs [77, 78]. Moreover, the addition of 1.5 % citric acid did not affect the severity or incidence of scouring after a postweaning *E. coli* challenge [78].

### Propionic acid

Propionic acid is an oily liquid and has disagreeable rancid odour. It is produced by *Propioniobacterium* in the manufacture of cheese and is also one of the major end products of bacterial fermentation. In an experiment with piglets, [79] added Luprosil-NC (product contains 53.5 % propionic acid) at levels of 0.3 and 1 %. Luprosil-NC did not affect pH, lactic acid concentration or SCFA concentration in the stomach and small intestine, but decreased *E. coli* counts in the stomach at concentrations of 1 % and not at 0.3 %. Sutton *et al.* [82] studied the effect of adding 0.25 % Luprosil-NC or 0.3 % sodium propionate on short chain fatty acids (SCFA) concentration and *Lactobacilli* and *E. coli* counts in digesta from the stomach, duodenum, caecum and colon in 8 weeks-old piglets. Addition of the organic acid did not significantly affect any of the parameters measured. Mathew et al. [80] added 0.25, 0.5 or 1 % Luprosil-NC to a control diet, and measured pH, numbers of *E. coli* and *Lactobacilli* in stomach, duodenum, caecum and colon in 8 and 12 week-old piglets. No effect of addition of the propionic acid-containing product was observed in 8 week-old piglets but 12 weeks-old piglets fed Luprosil-NC showed higher *Lactobacilli* counts in the duodenum than those fed the control diet. EFSA [81] have reported that the maximum safe limit of Propionic acid for poultry is 10 g/kg complete diet and for pigs it is 30 g/kg complete diet. The corresponding safe concentrations in water for drinking would be 4 and 10 g per litre, respectively. They stated that Propionic acid, Sodium propionate and calcium propionate are authorized in EU for use in food.

### Fumaric acid

Addition of fumaric acid at 1.5 % level had no influence on pH, VFA concentration, and microflora (counts of total anaerobe bacteria, *Lactobacilli, Clostridia*, and *E. coli*) in the entire GI-tract [77, 78] in weanling piglets. The concentration of fumaric acid in the stomach and jejunum was increased when a control diet was supplemented with 1.5 % fumaric acid [77, 78]. The acid did not affect the density of *Lactobacilli* or *E. coli* along the GI-tract. Sutton et al. [82] added 0.3 % Na-fumarate to a control diet, but did not see any significant effect of the acid on the concentration of SCFA and the density of *Lactobacilli* or *E. coli* along the GI-tract. The same authors observed a decreasing effect of 1 % fumaric acid on *E. coli* counts in the stomach of 8 week-old piglets, and an increasing effect on VFA in the caecum compared to a control diet. No effect on VFA concentration, *Lactobacilli* counts along the GI-tract or on *E. coli* in the duodenum, caecum, or colon was detected. Studies by [83] demonstrated a significant decreasing effect of 1.8 % fumaric acid on *Lactobacilli* in duodenum, jejunum, ileum, caecum and colon; of eubacteria in duodenum, jejunum and ileum; of enterococci in duodenum and jejunum; and of *E. coli* in the jejunum of 10 week-old piglets. Gabert and Sauer [84] fed diets containing 1.5 or 3 % fumaric acid or 1.5 % Na-fumarate to ileal canulated weaners. There was no effect of diet on the pH of the ileal digesta. There was a tendency for higher concentration of total SCFA in the animals fed the experimental diets as compared to animals fed the control diet.

### Lactic acid

This acid is a natural constituent of some feed stuffs and also is produced by many bacteria like *Lactobacillus*, *Streptococcus, Bifidibacterium* etc. The addition of lactic acid in concentrations of 0.8 % to a control weaner diet effectively reduced the levels of *E. coli* in the duodenum and jejunum of 8 weeks old piglets [23]. Thompson and Lawrence [18] measured a lower gastric pH when 1 % lactic acid was added to drinking water and offered to gastric cannulated piglets. Furthermore, lactic acid delayed the multiplication of an enterotoxigenic *E. coli* and reduced the mortality rate of the animals. The supplementation of milk with 1 % lactic acid resulted in lower counts of coliform bacteria and *Lactobacilli* in the stomach and duodenum of 2 weeks-old weaned pigs as compared to normal milk. Piglets fed diets supplemented with 0.7, 1.4 or 2.8 % lactic acid also showed changes of gastrointesinal characteristics [85]. The pH in the GI-tract was reduced by the acid addition and the *Lactobacilli* density was reduced in the small intestine (1.4 % lactic acid) and higher in the caecum and colon (0.7 % lactic acid) of pigs fed the diet added lactic acid.

### Formic acid

Formic acid is a colourless, transparent liquid with pungent odour. Bolduan et al. [79] studied the effect of adding 0.35 or 1.2 % formic acid to piglets, and observed a lower pH in the stomach following addition of 0.35 % formic acid with no effect on SCFA concentration along the GI-tract. Roth et al. [86] fed diets supplemented with 0.6, 1.2, 1.8 or 2.4 % formic acid to weanling pigs and analysed digesta from the stomach, small intestine, caecum and colon. The addition of formic acid resulted in higher pH values in the contents of small intestine, caecum and colon. Furthermore, the concentration of lactic acid in the small intestine and the concentration of SCFA in the colon were lower as compared to animals fed the control diet. In a similar experiment with piglets, [83] observed higher numbers of coliform bacteria in the duodenum (1.8 % formic acid), lower *Lactobacilli* and coliform counts in the caecum and colon, and lower numbers of eubacteria in the caecum as compared to pigs fed a control diet.

Maribo et al. [85] fed piglets diets supplemented with 0.7 or 1.4 % formic acid. Addition of 1.4 % formic acid reduced the pH in the stomach, caecum and colon, with lower concentrations of lactic acid in the small intestine and higher lactic acid concentrations in the colon The concentration of formic acid in the stomach, that of acetic acid in the small intestine, and that of acetic acid and propionic acid in caecum and colon was higher in acid supplemented animals. Furthermore, the authors found lower numbers of lactobacilli in distal small intestine and caecum, lower coliform counts in the stomach (0.7 % formic acid) and lower yeast counts along the GI-tract. The addition of Potassium diformate at concentrations of 1.8 % to a weaner diet did not significantly affect the pH along the GI-tract, but increased the concentration of formic acid in the stomach and small intestine. A decreased number of total anaerobe bacteria, lactic acid bacteria and yeast were found along the GI-tract. The intestinal counts of coliforms were numerically but not significantly reduced [38]. Using the same product at a dose of 1.2 %, [63] observed a decreasing effect of the acid on the number of coliform bacteria in the duodenum, jejunum and rectum of growing finishing pigs. On the other hand, [87] observed lowered pH of duodenal digesta in piglets up to 65 h post-feeding of potassium diformate (0.9 and 1.8 %) [88] fed piglets with 0.9 or 1.8 % Potassium diformate and observed a reduction of pH, number of coliforms and *Streptococci* in the stomach, and a reduction of coliforms in the colon. No effect on *Lactobacilli* in any segment of the GI-tract was detected.

### Benzoic acid

Though this acid is not approved as as an additive or preservative the supplementation of feed with benzoic acid resulted in significantly lower counts of lactic acid bacteria*, Lactobacilli* and yeast throughout the entire GI- tract [85]. Benzoic acid could be detected in considerable amounts in the stomach and in smaller amounts in the small intestine, indicating that benzoic acid may not be metabolised as fast as other organic acids.

## Conclusion

The modern livestock enterprises are like a tussel between consumer’s concern on animal and human health with an increasing demand for animal products. Organic acids and their salts were proven as potential growth promoters in weaned piglets, finishing pigs and pregnancy sows. They can also be used safely and effectively with other additives and so these are better accepted by the feed manufacturers, animal producers and public. The main mode of action of organic acids is through their antimicrobial effects, the magnitude of which is dependent on the chemical properties of the individual acid or acid salt. Several investigations have shown a strong bactericidal effect of organic acid without significantly decreasing the pH-value in the GI-tract. Organic acids, especially butyrates and propionates also act by stimulating secretion of pancreatic enzymes. However, exact modes of action of the organic acidifiers are still to understood and in particular their action in different sections of the gastro-intestinal tract is still unclear. In a nutshell, organic acids can stimulate secretion of pancreatic enzymes, lower gastric pH, inhibit pathogens, acts as an energy source during GI-tract intermediary metabolism, improves mineral utilization by chelation process, enhances apparent total tract digestibility and improves growth performance.
